# Skeletal muscle‐specific over‐expression of the nuclear sirtuin SIRT6 blocks cancer‐associated cachexia by regulating multiple targets

**DOI:** 10.1002/rco2.27

**Published:** 2020-12-23

**Authors:** Sadhana A. Samant, Vinodkumar B. Pillai, Mahesh P. Gupta

**Affiliations:** ^1^ Department of Surgery University of Chicago 5841 South Maryland Avenue Chicago IL 60637 USA; ^2^ Committee on Molecular Medicine and Pathology, Pritzker School of Medicine University of Chicago Chicago IL USA

**Keywords:** Cachexia, Sirtuins, SIRT6, Skeletal muscle, Muscle wasting

## Abstract

**Background:**

During cancer cachexia, cytokines released from tumour cells can alter body's metabolism, which can lead to onset of this disease process. Biological basis of cachexia is multifactorial; hence, it is important to identify and modulate multiple targets to curtail the process of cachexia. Previously, we reported that the nuclear sirtuin, SIRT6, blocks expression of myostatin, a negative regulator of muscle growth, through modulation of the NF‐κB signalling. This study was undertaken to test whether muscle‐specific over‐expression of SIRT6 can block the cancer‐associated muscle wasting *in vivo* and to identify additional relevant targets of SIRT6, which can explain its ability to maintain muscle health.

**Methods:**

We generated a skeletal muscle‐specific SIRT6 over‐expressing transgenic mouse line (Sk.T6Tg) expressing SIRT6 at a moderate (two‐fold to four‐fold) level, compared with its control littermates. To generate a cancer‐cachexia model, B16F10 mouse melanoma cells were injected subcutaneously in the flanks of mice. Gastrocnemius muscle tissues from non‐tumour and tumour controls and Sk.T6Tg mice (*n* = 5–20) were analysed by histology, immunoblotting, and RT‐qPCR. Plasma samples of mice were evaluated using cytokine arrays and ELISA in both non‐tumour and tumour conditions.

**Results:**

Our results demonstrate dual benefits of muscle‐specific moderate over‐expression of SIRT6 in a mouse model of cancer‐cachexia. In tumour‐bearing mice, SIRT6 over‐expression preserved muscle weight (*P* < 0.001) and fibre size (*P* < 0.005) as well as suppressed tumour growth (*P* < 0.05). SIRT6 over‐expression significantly reduced myostatin expression and plasma free fatty acids levels but maintained plasma insulin levels in tumour‐bearing mice. These positive effects of SIRT6 were associated with downregulation of the circulatory chemokine, CXCL10, and the myokine, WNT4. SIRT6 also upregulated expression of GLUT4, the major glucose transporter in the skeletal muscle. These results for the first time demonstrate that SIRT6 regulates multiple targets to limit tumour growth and cancer‐associated muscle atrophy.

**Conclusion:**

Given the multifactorial nature of cachexia, SIRT6, which concurrently controls multiple pathways, can be a valuable therapeutic target to overcome this debilitating syndrome.

## Introduction

Cachexia is a syndrome associated with many terminal diseases and manifests as an involuntary wasting of skeletal muscle with or without loss of adipose tissue.^1^ Cachexia not only deteriorates the quality of life but also increases mortality in the cancer patients. It is reported to be prevalent in 50–80% of the advanced stage cancer patients, of which 20% succumb to death primarily due to the wasting syndrome and not because of the cancer itself.^2^ Cachexia is a multifactorial syndrome with a complex metabolic involvement, which manifests with many secondary symptoms such as inflammation, fatigue, and anaemia that worsens the prognosis of the primary disease.^3^ Although considerable advances are made in the last decade to develop new drugs to alleviate the devastating effects of cachexia, no approved therapy is yet available. Because this syndrome appears to be multimodal, it is appropriate to tackle it by controlling at many causative levels in the early stages of the disease. Moreover, it is important to identify a target, which can simultaneously modulate multiple pathways beneficially to overcome the muscle‐wasting syndrome.

Previously, it was observed that mice deficient in a chromatin‐bound lysine deacylase, SIRT6, exhibited a phenotype analogous to cachexia with loss of skeletal muscle and fat tissue as well as extensive inflammation and perturbed energy metabolism.^4^, ^5^ SIRT6 belongs to an evolutionarily conserved group of NAD^+^‐dependent enzymes called sirtuins, which can sense cellular energy and redox status. All the seven mammalian sirtuins (SIRT1–7), which are localized to different cellular compartments, control a wide range of cellular functions including metabolism, genome stability, growth, and aging.^6^ In relevance to cachexia, our recent study shows that SIRT6 was able to downregulate expression of myostatin (MSTN), a member of the transforming growth factor (TGF)‐β family and a known potent negative regulator of skeletal muscle mass.^5^ SIRT6 is recognized as an enzyme with multiple functions, influencing a wide range of diseases associated with cachexia such as cancer, diabetes, cardiovascular, and renal failure.^7^, ^8^ SIRT6 is demonstrated to play a key role in maintaining glucose and lipid homeostasis.^8^ There is evidence that in cachexia, these metabolic pathways are dysfunctional.^9^, ^10^, ^11^ In addition, inflammation is also a major contributor to the pathology of cachexia,^12^ and SIRT6 is a known suppressor of pro‐inflammatory transcription factors such as NF‐κB and c‐JUN.^13^, ^14^, ^15^ Furthermore, SIRT6 and NF‐κB are considered to be the two essential components of communication link between inflammation and metabolism.^16^ We have reported before that by targeting NF‐κB signalling, SIRT6 suppresses MSTN expression and thereby maintains normal levels of myogenic factors in muscle cells.^5^ SIRT6 has been demonstrated as a tumour‐suppressor and is shown to antagonize tumorigenesis by modulating multiple pathways. It is a key negative regulator of inflammation, metabolic reprogramming, and genomic instability; three major pathways implicated in cancer development.^17^, ^18^ In addition, another mechanism by which SIRT6 is proposed to act as a tumour suppressor is by defatty‐acylation of lysine in R‐RAS2, a member of *Ras* family of GTPases, frequently implicated in human cancers.^19^

Skeletal muscle is one of the most affected tissues in cachexia. Being one of the major organs in the body, maintenance of skeletal muscle mass and function is crucial to metabolic health of the body. Systemic stimuli such as stress, inflammation, and insulin resistance, emerging from diseases like cancer or diabetes, can perturb energy balance in skeletal muscle. Morbidity and mortality of cancer patients are directly correlated to muscle atrophy.^20^, ^21^ It has been demonstrated that muscle‐specific deficiency of SIRT6 results in impaired glucose metabolism, attenuated insulin‐sensitivity, and weakened tolerance to exercise.^22^ Similar dysfunctions are also observed in cachectic cancer patients.^23^ The ability of SIRT6 to act as a tumour suppressor and to help promote muscle health prompted us to explore whether skeletal muscle‐specific over‐expression of SIRT6 in mice could limit the destructive consequences of cancer‐associated cachexia. Hence, we generated skeletal muscle‐specific SIRT6 over‐expressing mice and studied its effect in tumour‐induced cachexia. Here, we report that over‐expressed SIRT6 regulates multiple targets to limit tumour progression and cancer‐associated muscle atrophy.

## Materials and methods

### Generation of skeletal muscle‐specific SIRT6 over‐expressing transgenic (Sk.T6Tg) mice and the tumour model

Floxed STOP‐*Sirt6* transgenic mice (InGenious Targeting Labs, NY) were generated as described earlier.^24^ Using offspring's tail DNA for PCR, germline incorporation of *Sirt6‐Flag* transgene was determined. PCR primers used were, forward: 5′‐TGCAACCCACAAAACATGAC‐3′ and reverse (complementary to the *Flag*‐tag sequence): 5′‐ACAATGCGATGCAATTTCC‐3′. Transgenic mice yield a PCR product of 454 bp.

Skeletal muscle‐specific SIRT6‐FLAG over‐expressing mice (Sk.T6Tg) were generated by breeding the ROSA26‐Floxed STOP‐*Sirt6‐Flag* mice with *Myl* ^1tm1(cre)sjb^/J mice (Jackson Laboratory, stock number 024713) that express CRE Recombinase from the skeletal muscle‐specific myosin light chain polypeptide 1 (*Myl1*) promoter. To genotype *Cre* ^+^ progeny, following set of primers was used, and PCR was carried out for tail DNA as per Jackson laboratory's instructions. Primer#25540: (5′‐CACACTGCTCTTCCAAGTGTC‐3′); Primer#25541: (5′‐AGTTACCTTAATAGCAGACAGATCG‐3′) and Primer#oIMR1709: (5′‐GCAAACGGACAGAAGCATTT‐3′). PCR products generated are 200 bp for wild type, 280 bp for mutant, and both for the heterozygous progeny. *Cre*‐mutant mice express the Cre recombinase in skeletal muscle tissue. Sk.T6Tg mice yield a 454 bp (*Sirt6‐Flag*‐specific) and a 280 bp (*Cre*‐mutant‐specific) PCR products. The University of Chicago Institutional Animal Care and Use committee (IACUC) reviewed and approved all the animal protocols.

Two‐month‐old male, littermate controls (CN, *n* = 20) and skeletal muscle‐specific SIRT6 over‐expressing (Sk.T6Tg, *n* = 10) mice were subcutaneously injected in the flank with B16F10 mouse melanoma cells. Cells (1 × 10^6^ in DMEM) were mixed in equal parts with matrigel membrane matrix (CB‐354248, ThermoFisher, USA) before injecting. Final injection volume was 100 μL, which contained 1 × 10^6^ cells. Plasma and tissues were harvested 15 days after injection for further analyses. All animal experiments were performed as per the relevant guidelines and regulations of IACUC and biosafety committee of the University of Chicago.

#### RNA extraction and real‐time RT‐qPCR

Total RNA was extracted from mouse gastrocnemius muscle tissue using TRIzol reagent (Invitrogen, CA) as per manufacturer's instructions. DNase I treatment, further purification of DNased‐RNA, and first strand cDNA synthesis were carried out as described previously.^5^ Using reverse‐transcribed (RT) cDNA and iQ SYBR Green mix (BioRad), RNA expression for targets was evaluated by real‐time qPCR. Sequences for the primers used in this study are tabulated in *Table* *1*. qPCR data for targets were normalized to RNA polymerase 2 expression in each sample, and comparative threshold cycle method (2^−ΔΔCt^) was used to calculate fold‐changes in gene expression.^25^

**Table 1:** Primer sequences used in this study

Mouse primers	5′‐3′ sequence
*Sirt6*F	ATG TCG GTG AAT TAT GCA GCA
*Sirt6*R	GCT GGA GGA CTG CCA CAT TA
*Wnt4*F	AGA CGT GCG AGA AAC TCA AAG
*Wnt4*R	GGA ACT GGT ATT GGC ACT CCT
*Cxcl10*F	CCA AGT GCT GCC GTC ATT TTC
*Cxcl10*R	GGC TCG CAG GGA TGA TTT CAA
*RNA Pol2*F	CTA AGG GGC AGC CAA AGA AAC
*RNA Pol2*R	CCA TTC AGC ATA CAA CTC TAG GC

#### Blood plasma analysis

Blood from mice was collected in EDTA coated tubes, which also contained EDTA to a final concentration of 5 mM as an anticoagulant. Plasma was separated by centrifugation at 2000×*g* for 15 min; supernatant was dispensed in smaller aliquots and stored at −80 °C until further use. Plasma insulin levels were estimated in 5 μL of samples using mouse Insulin ELISA kit (Crystal Chem) as per manufacturer's instructions. Free fatty acid contents were estimated for 10 μL plasma samples, using the colorimetric kit from Biovision Inc. (K612–100). Estimation was carried out according to manufacturer's protocol. WNT4 concentration in plasma was estimated using 10–20 μL plasma and mouse protein WNT4 ELISA kit (MyBioSource Inc., MBS903402) as per manufacturer's instructions. For proteome profiler assay (R&D systems), 100 μL of plasma samples were used on mouse cytokine array panel A (ARY006). Assay was carried out as per the manufacturer's instructions. This array contains antibodies for 40 different cyto/chemokines blotted in duplicate.

#### Histology and western blotting

Gastrocnemius muscles were fixed in neutral formalin. For tumour‐bearing mice, non‐tumour side muscle was used. The HTRC core facility of the University of Chicago sectioned the fixed tissues and stained them with haematoxylin–eosin stains. Imaging and quantitation were carried out using Perkin Elmer's Pannoramic Scanner with Pannoramic viewer software (3dhistec Ltd., USA). Tissue lysates for gastrocnemius muscle from non‐tumour side were prepared using RIPA buffer, and expression levels of proteins were checked by immunoblotting 25–30 μg of lysates with indicated antibodies as previously described.^5^ Total protein loading was checked by Coomassie staining of blots using GelCode blue stain (ThermoFisher). Sources of antibodies used in this study are provided in *Table* *2*. Immunoblots were quantified using Image J (NIH) and Quantity one (BioRad) software.

**Table 2:** Antibodies used in this study

Antibody name	Company	Catalogue number
Mouse GLUT‐4	Cell Signalling Technology	2213
Rat myostatin	R&D Systems	MAB788
HRP‐β‐actin	Santa Cruz Biotechnology	sc47778HRP
Rabbit SIRT6	Cell Signalling Technology	12486
Rat WNT‐4	R&D Systems	MAB4751
Mouse α‐tubulin	Santa Cruz Biotechnology	sc32293
Anti‐rabbit HRP conjugate	Cell Signalling Technology	7074
Anti‐mouse HRP conjugate	Cell Signalling Technology	7076
Anti‐rat HRP conjugate	Santa Cruz Biotechnology	sc2065

#### Tumour assessment

Tumour size was measured using Vernier calliper, and tumour volume was calculated using the formula: V (mm^3^) = (π/6) × L × W × H; where V is volume, L is length, W is width, and H is height of the tumour.^26^

#### Statistical analysis

Graphical data are presented as mean ± standard error of means (SEM). To determine statistical significance between two groups, Student's *t*‐test was used.

## Results

### Generation and validation of skeletal muscle‐specific SIRT6 over‐expressing transgenic (Sk.T6Tg) mice

**Figure 1:**
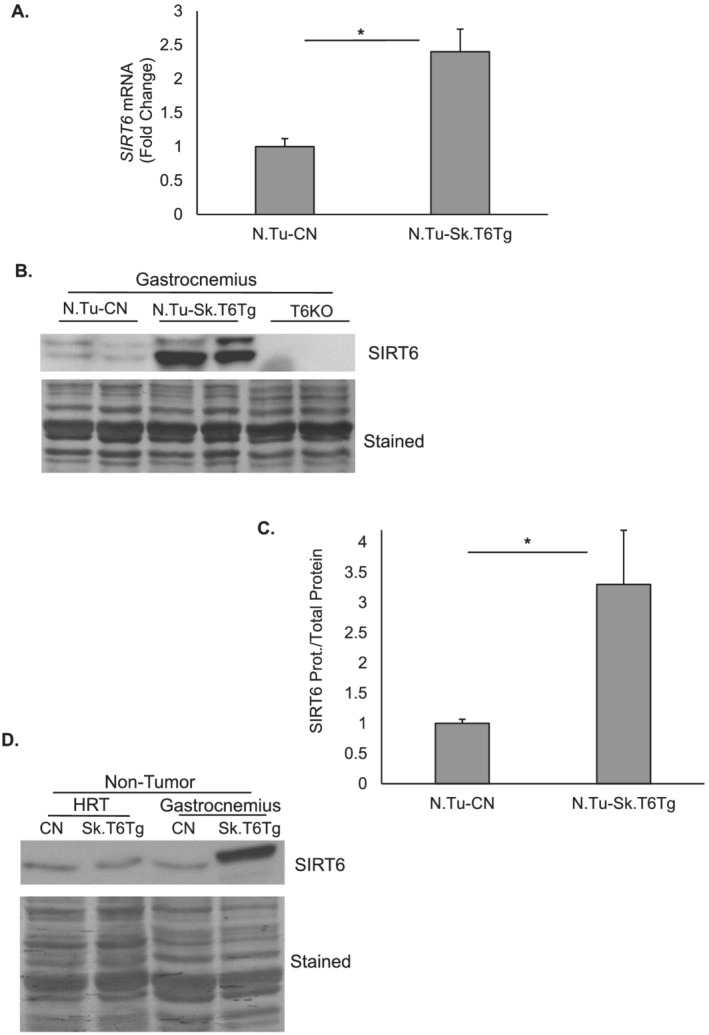
Characterization of the skeletal muscle‐specific SIRT6 over‐expressing transgenic (Sk.T6Tg) mouse line in non‐tumour (N.Tu) setting. (*A*) Bar graph showing mRNA levels of *Sirt6* normalized to *RNA polymerase 2* expression between the N.Tu‐CN and N.Tu‐Sk.T6Tg mice. (*B*) Western blot showing SIRT6 expression levels in gastrocnemius muscle of N.Tu‐CN, N.Tu‐Sk.T6Tg, and SIRT6 knock‐out (T6KO) mice. (*C*) Bar graph presenting quantitation of the SIRT6 protein normalized to total protein per lane. (*D*) Immunoblot showing skeletal muscle‐specific SIRT6 over‐expression for N.Tu‐CN and N.Tu‐Sk.T6Tg mice. Coomassie blue‐stained blots are shown as loading controls. For each experiment, five to seven mice were used. *P < 0.05. Data represented as mean ± SEM. CN, control; HRT, heart; SEM, standard error of the mean; Sk., skeletal muscle.

To generate mice that over‐expressed SIRT6 at moderate level, only in skeletal muscle, we first knocked‐in the C‐terminal FLAG‐tagged mouse *Sirt6* cDNA with a Floxed‐STOP cassette in the mouse ROSA26 genomic locus. To over‐express SIRT6‐FLAG specifically in the skeletal muscle cells from the extra copy of cDNA under the control of ROSA26 promoter, homozygous mice were bred with mice expressing Cre‐recombinase under the control of myosin light chain (*Myl*) promoter. Schematic representation for the generation of these mice is provided in the Supporting Information, *Figure* S1A. Littermates lacking the Cre‐recombinase expression were used as the controls (CN), which express SIRT6 at endogenous level. Sk.T6Tg mice were born in normal Mendelian ratio without any embryonic lethality or any growth or morphological abnormalities when compared with their control littermates. A representative agarose gel (Supporting Information, *Figure* S1B) shows genotyping of these mice, using the FLAG‐tag sequence‐specific reverse primer as one of the PCR primers, to identify the transgenic copy of *Sirt6* cDNA. At mRNA level, *Sirt6* expression in over‐expressing mice was about two‐fold to 2.5‐fold that of control mice (*Figure* 1A). As seen in *Figure* 1B and 1C, expression level of SIRT6 protein in the gastrocnemius muscle was about 2.5–3.5 times that of endogenous controls. Specificity of SIRT6 protein bands was validated by running protein lysates prepared from the gastrocnemius muscles of whole body *Sirt6* knock‐out (T6KO) mice on the same immunoblot (*Figure* 1B). In addition, skeletal muscle‐specific SIRT6 over‐expression was confirmed by analysing protein lysates from heart (HRT) tissue along with gastrocnemius muscle (*Figure* 1D). All the analyses presented in aforementioned experiments are carried out for tissue samples collected from non‐tumour (N.Tu)‐bearing mice.

### Dual benefits of muscle‐specific over‐expression of SIRT6 in tumour‐bearing mice

**Figure 2:**
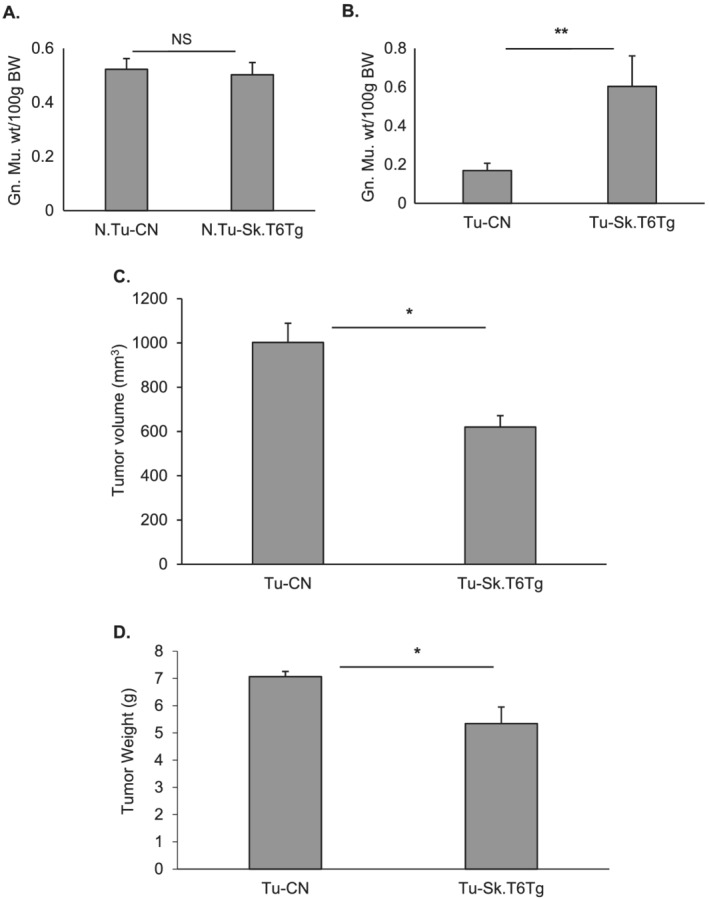
Muscle‐specific over‐expression of SIRT6 benefits in two ways. Bar graphs showing comparative weight of gastrocnemius muscle (Gn. Mu.) normalized to body weight for (*A*) N.Tu‐CN and N.Tu‐Sk.T6Tg mice (*n* = 7–10) and (*B*) in tumour‐bearing mice (Tu‐CN vs. Tu‐Sk.T6Tg) (*n* = 10–20). Over‐expressed SIRT6 not only preserves muscle weight, it also reduces (*C*) tumour volume and (*D*) tumour weight significantly. Data represented as mean ± SEM, *n* = 10–20 mice, **P < 0.001, *P < 0.05. BW, body weight; NS, non‐significant.

**Figure 3:**
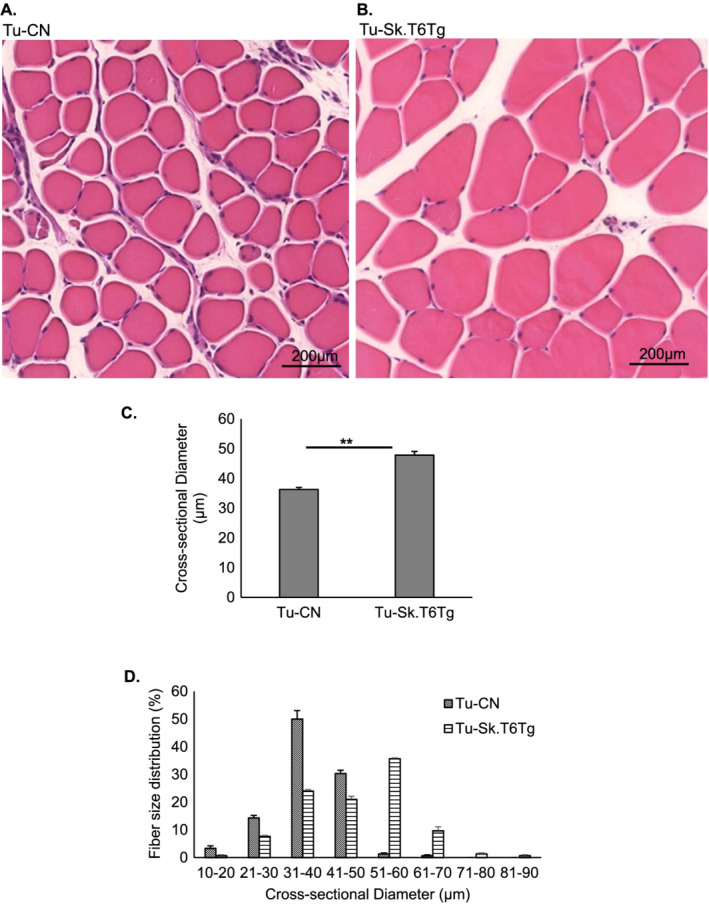
Skeletal muscle‐specific over‐expression of SIRT6 impedes cancer‐induced muscle degeneration. Representative images showing histology of gastrocnemius muscle sections stained with haematoxylin–eosin for (*A*) Tu‐CN and (*B*) Tu‐Sk.T6Tg mice. Scale bar: 200 μm. Bar graphs showing (*C*) muscle cross‐sectional diameter and (*D*) fibre distribution grouped in ascending order of 10 μm‐apart size classes. Values in panel (*D*) are represented as percentage (%) of total number of fibres counted for the two aforementioned mice categories. Data represented as mean ± SEM, *n* = 3–5 mice per group. **P < 0.005.

We have previously observed that mice deficient in SIRT6 show degenerative muscle phenotype, suggesting that activation of SIRT6 could protect the muscle from undergoing atrophy.^5^ Because muscle atrophy is a major hallmark of cancer‐associated weight loss, we challenged the muscle‐specific SIRT6 over‐expressing (Sk.T6Tg) mice with subcutaneous injection of B16F10 melanoma cells on the flank of the animal. These tumour cells are syngeneic to the C57BL6 background of the SIRT6 over‐expressing and littermate mice used in this study. Mice developed solid melanoma tumours by 15 days, and approximately 10% mortality was observed in control tumour‐bearing mice (Tu‐CN), an observation similar to that reported by earlier studies.^27^, ^28^ On the contrary, we observed no mortality for tumour‐bearing SIRT6 over‐expressing (Tu‐Sk.T6Tg) mice in these 2 weeks period, at which time these mice were sacrificed for further analyses. B16F10 melanoma is reported to cause cachexia and reduce locomotor activity with impaired skeletal muscle strength in mice.^28^ For all experiments with tumour‐bearing mice, we used the gastrocnemius muscle (Gn.Mu.) harvested from the non‐tumour‐bearing side hind limb to avoid a direct physical effect of the growing tumour mass on the flank. In non‐tumour conditions, weight of gastrocnemius muscle between control (N.Tu‐CN) and SIRT6 over‐expressing (N.Tu‐Sk.T6Tg) mice did not differ significantly when normalized to body weight (*Figure* 2A). However, in tumour environment, we observed that when normalized to initial body weight, gastrocnemius muscle weight of Tu‐CN mice showed significant reduction compared with that for Tu‐Sk.T6Tg mice (*Figure* 2B). SIRT6 over‐expression not only helped in preserving the muscle weight, but it also significantly reduced tumour volume (*Figure* 2C) and tumour weight (*Figure* 2D). Our results are in agreement with previous reports, which show SIRT6 as an inhibitor of tumorigenesis.^17^, ^18^, ^19^ We also measured the cross‐sectional diameter (CSD) of gastrocnemius muscle fibres in Tu‐CN and Tu‐Sk.T6Tg mice. There was a significant decline in the diameter of muscle fibres of Tu‐CN mice, compared with that in Tu‐Sk.T6Tg mice (*Figure* 3A–3C). Contrary to this finding, we did not observe any significant change in gastrocnemius muscle fibre diameter with SIRT6 over‐expression in non‐tumour condition (Supporting Information, *Figure* S2A–S2C). An earlier study in humans has noted a 25% reduction in the mean muscle fibre diameter in cachectic subjects with low muscularity compared with the non‐cachectic controls.^29^ Decrease in muscle fibre size hampers the force generation ability of muscle.^30^ We also determined range of fibre size distribution in gastrocnemius muscle, by performing small Feret diameter analysis. This analysis revealed that in Tu‐Sk.T6Tg mice, fibres of higher size diameters (51–90 μm) were preserved, when compared with those in Tu‐CN mice (*Figure* 3D). In non‐tumour condition, SIRT6 over‐expression did not alter fibre size range distribution (Supporting Information, *Figure* S2D) unlike in tumour environment. Overall, our results indicated that SIRT6 over‐expression in skeletal muscle not only reduced tumour progression but also blocked cancer‐induced muscle degeneration.

### SIRT6 over‐expression blocks cancer‐associated muscle atrophy by mitigating metabolic dysregulations

**Figure 4:**
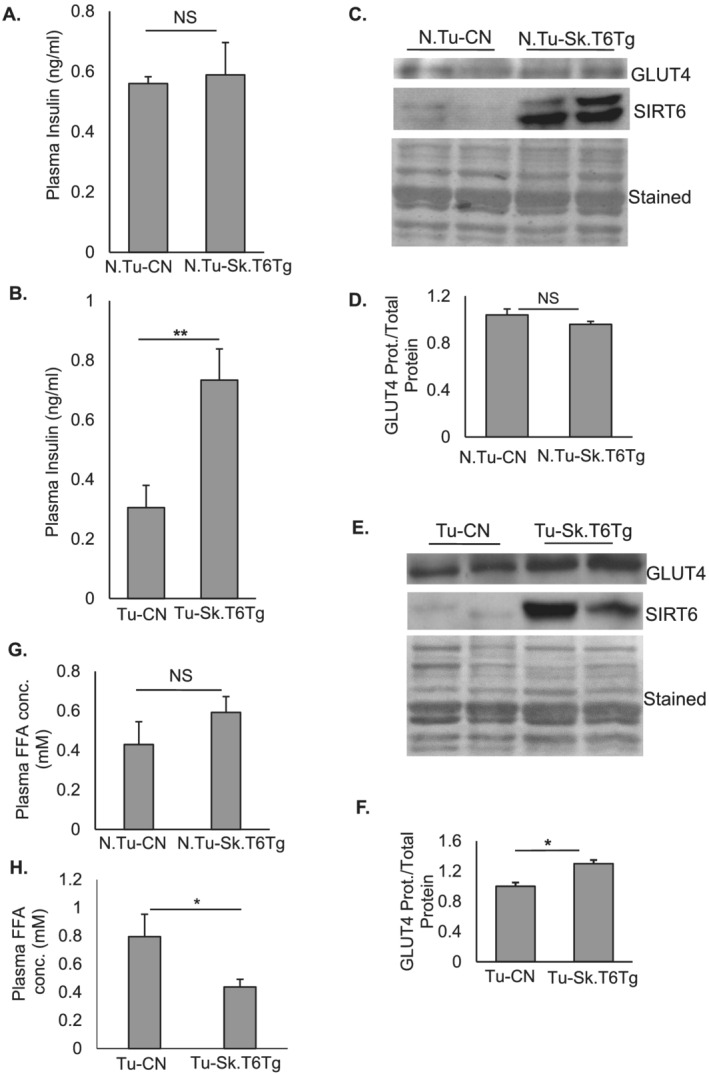
Skeletal muscle‐specific over‐expression of SIRT6 regulates plasma insulin levels in tumour‐bearing mice. (*A*) Plasma insulin levels for N.Tu‐CN and N.Tu‐Sk.T6Tg mice (*n* = 7–10). (*B*) in tumour environment, SIRT6 over‐expression upregulates plasma insulin levels for Tu‐Sk.T6Tg compared to Tu‐CN mice (*n* = 10–20). Representative western blots and their quantitation showing GLUT4 protein expression in gastrocnemius muscles of N.Tu‐CN with N.Tu‐Sk.T6Tg (*C* and *D*) and Tu‐CN with Tu‐Sk.T6Tg mice (*E* and *F*) respectively. GLUT4 protein level is normalized to the total protein loaded (Coomassie‐stained blots). (*G*) Plasma FFA concentration estimated for N.Tu‐CN and N.Tu‐Sk.T6Tg mice (*n* = 7–10). (*H*) SIRT6 significantly downregulated circulating free‐fatty acid levels in Tu‐Sk.T6Tg compared with Tu‐CN mice (*n* = 10–20). Data represented as mean ± SEM. NS, non‐significant. *P < 0.05, **P < 0.005.

SIRT6 is an established regulator of glucose and lipid metabolism.^8^ Interestingly, these are also major metabolic pathways perturbed in cancer‐associated cachexia.^31^ In cachectic cancer patients, it was reported recently that serum insulin level is significantly reduced.^27^ In our model, when we measured plasma insulin levels in mice fed *ad libitum*, we found no significant change in insulin levels of SIRT6 over‐expressing mice (N.Tu‐Sk.T6Tg) and their control littermates (N.Tu‐CN) (*Figure* 4A). However, in tumour‐bearing mice, plasma insulin levels were significantly higher in Tu‐Sk.T6Tg mice, compared with Tu‐CN animals (*Figure* 4B). We therefore posit that although SIRT6 was over‐expressed locally in skeletal muscle tissue, it could indirectly overcome the reduction in plasma insulin levels through a paracrine effect. Insulin supplementation has been demonstrated to slow tumour progression in mice carrying B16F10 melanoma,^27^ suggesting that reduced tumour growth seen in our SIRT6 over‐expressing mice could be attributed partly to upregulation of insulin levels in these mice.

Skeletal muscle, which constitutes 40% of the body mass, is one of the major glucose utilizing tissues of the body.^32^, ^33^ The key regulator for transporting insulin‐dependent glucose in skeletal muscle cells is glucose transporter 4 (GLUT4).^33^ GLUT4 protein expression in gastrocnemius muscle of N.Tu‐CN and N.Tu‐Sk.T6Tg mice did not change significantly (*Figure* 4C and 4D); however, in tumour‐bearing mice, GLUT4 was significantly upregulated with SIRT6 over‐expression (*Figure* 4E and 4F). Reduced GLUT4 expression was previously reported in skeletal muscle tissue of type 2 diabetic patients with insulin‐resistance.^34^ Given that GLUT4 is an insulin‐responsive glucose transporter, the increased GLUT4 level seen in our tumour model could be either because of the direct effect of SIRT6 on *Glut4* promoter or a consequence of increased plasma insulin levels or both.^35^ Although at this point, we cannot precisely predict on how SIRT6 promotes expression of GLUT4 in muscle, our results suggest that SIRT6 over‐expression in skeletal muscle likely mitigates cancer‐induced cachexia by preserving whole body glucose homeostasis.

Additionally, levels of circulating free fatty acids (FFAs) are reported to be high in cachectic cancer patients.^36^, ^37^ In our non‐tumour animals, we did not detect any significant change in the plasma FFA levels between N.Tu‐CN and N.Tu‐Sk.T6Tg mice (*Figure* 4G), consistent with a previous report where FFA levels were analysed in another mouse model of SIRT6 over‐expression.^38^ Conversely, SIRT6 over‐expression in tumour‐bearing mice significantly down‐regulated plasma FFA levels (*Figure* 4H). Insulin administration is shown to lower plasma FFA levels in cachectic tumour‐bearing rats.^39^ Taken together, these results indicated that in our mouse model of cancer‐cachexia, skeletal‐muscle specific over‐expression of SIRT6 increased plasma insulin levels, which likely contributed to reduce tumour growth and to preserve muscle mass.

### Identifying diverse SIRT6 targets, which help to maintain skeletal muscle mass

**Figure 5:**
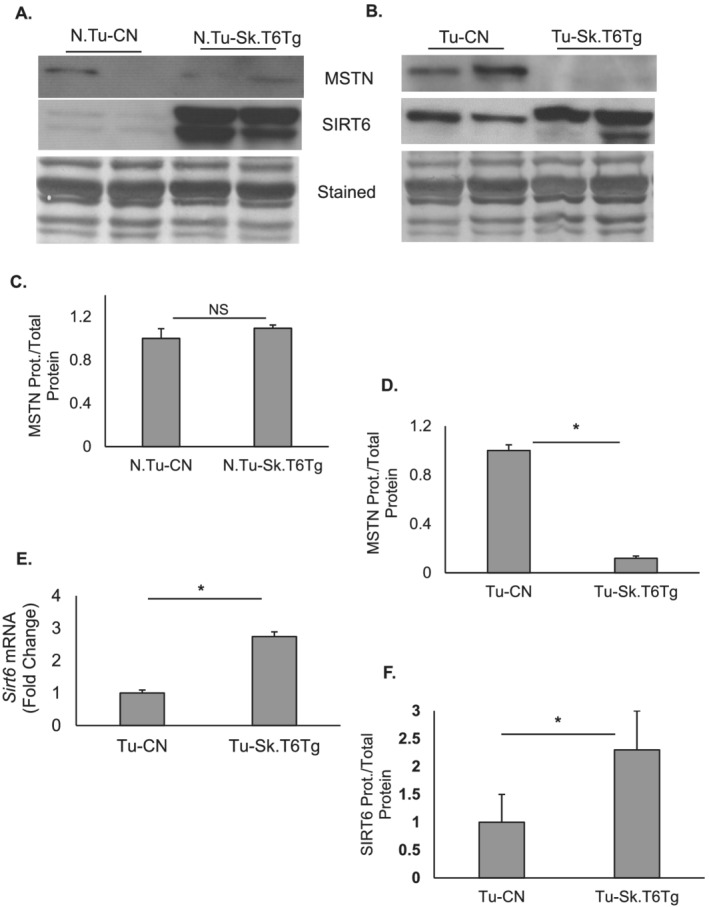
SIRT6 over‐expressed in skeletal muscles modulates myostatin expression in tumour environment. (*A*) Representative western blots showing protein levels of myostatin (MSTN) and SIRT6 in gastrocnemius muscle for the control, and transgenic mice with non‐tumour (*A*) and tumour background (*B*). Bar graphs presenting quantitation (*n* = 5–8 mice) of MSTN expression normalized to total protein loaded for samples in (*C*) non‐tumour and (*D*) tumour setting. (*E*) Bar graph shows comparative mRNA level of *Sirt6* in Tu‐CN and Tu‐Sk.T6Tg mice (*n* = 5–7 mice). (*F*) Quantitation of SIRT6 protein expression for sample groups shown in panel (*B*) (*n* = 8–12 mice). Bar graphs represent data as mean ± SEM. NS, non‐significant, *P < 0.05.

**Figure 6:**
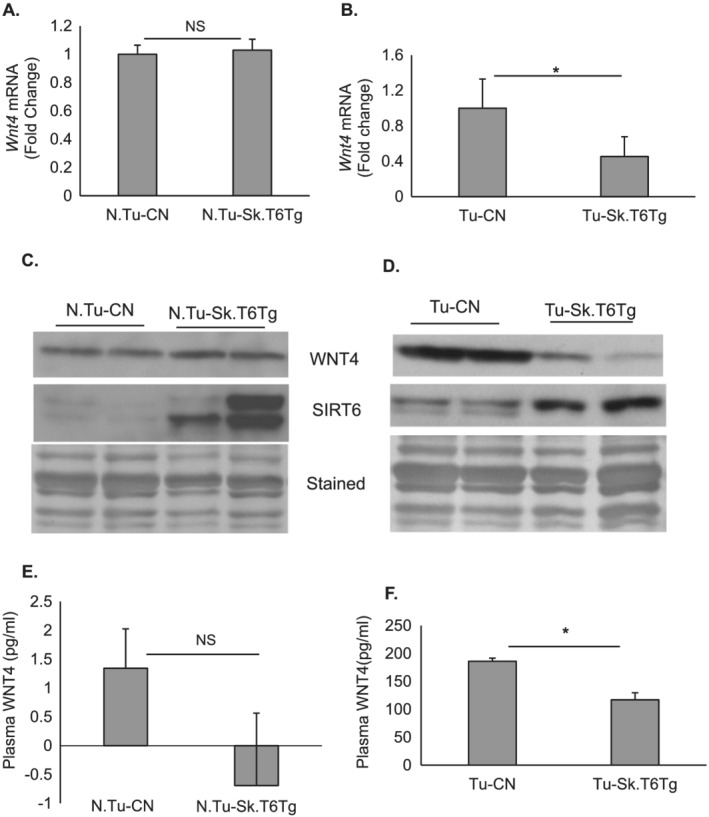
Over‐expressed SIRT6 also targets WNT4 in gastrocnemius muscle. (*A*) no significant change in *Wnt4* mRNA expression in non‐tumour background but (*B*) SIRT6 downregulates *Wnt4* (normalized to *RNA polymerase 2*) mRNA expression in tumour setting (*n* = 5–7 mice). Representative western blots showing WNT4 protein expression for both mice categories in non‐tumour (*C*), and (*D*) tumour‐bearing conditions (*n* = 5–8). Coomassie blue‐stained blots are shown as loading controls. Bar graphs showing plasma WNT4 concentrations for non‐tumour (*E*) (*n* = 7–10), and tumour‐bearing (*F*) control and Tg mice (*n* = 10–20 mice). Bar graphs represent data as mean ± SEM. NS, non‐significant. *P < 0.05.

Skeletal muscle is recognized as an endocrine organ.^40^ Accumulating evidence suggest that muscle‐secreted factors like myokines, miRNAs, and metabolites can influence functions of pancreatic β‐cells.^41^ We sought out to identify possible targets through which muscle‐specific SIRT6 over‐expression might exert a paracrine effect to normalize perturbed metabolism in cancer‐associated cachexia. In line with our previous report,^5^ we noticed that in tumour‐bearing mice, SIRT6 over‐expression significantly downregulated expression of myostatin (MSTN), a negative regulator of muscle mass, whereas in non‐tumour‐bearing mice, no significant change was observed (*Figure* 5A–5D). We also examined *Sirt6* mRNA and protein levels in gastrocnemius muscle samples of tumour‐bearing mice. The SIRT6 expression levels (*Figure* 5E and 5F) in tumour‐bearing mice were comparable with those seen in non‐tumour conditions (*Figure* 1A–1C), suggesting that tumour environment has not significantly altered SIRT6 expression in these mice. SIRT6 is a chromatin‐associated transcriptional repressor protein.^42^ We therefore determined additional SIRT6 targets, which could explain how muscle‐specific SIRT6 over‐expression regulates plasma insulin levels in a paracrine manner. An earlier study has found that WNT4, a secreted glycoprotein and a known inhibitor of WNT signalling pathway, is released from skeletal muscle and adipose tissue.^43^ WNT4 plays crucial role as a communicator between pancreatic β‐cells and peripheral insulin‐responsive tissues such as skeletal muscle.^43^ Kozinski and colleagues have reported that WNT4 levels are significantly increased in the muscle tissue and blood plasma of diabetic rats, and downregulation of WNT signalling caused due to WNT4 upregulation is shown to cause β‐cell dysfunction and reduced insulin secretion.^43^ Multiple studies have shown a cross talk between WNT and insulin signalling and have demonstrated that activation of WNT signalling increases insulin sensitivity of skeletal muscle cells.^44^, ^45^, ^46^ We therefore measured WNT4 expression in our model of cachexia. In non‐tumour mice, SIRT6 over‐expression did not influence *Wnt4* mRNA expression (*Figure* 6A); however, in tumour‐bearing mice, gastrocnemius muscles of Tu‐Sk.T6Tg mice showed a significant downregulation of *Wnt4* mRNA levels, compared with that in Tu‐CN mice (*Figure* 6B). Change in *Wnt4* mRNA levels also reflected in its protein expression. While there was no significant change in WNT4 protein expression in gastrocnemius muscle of N.Tu‐CN and N.Tu‐Sk.T6Tg mice (*Figure* 6C and Supporting Information, *Figure* S3A), in tumour‐bearing mice, SIRT6 over‐expression significantly reduced its expression in the muscle (*Figure* 6D and Supporting Information, *Figure* S3B). Similarly, plasma levels of WNT4 did not change significantly with SIRT6 over‐expression in the non‐tumour environment and were barely detectable (*Figure* 6E). However, with tumour background, upregulated WNT4 plasma levels were significantly downregulated in Tu‐Sk.T6Tg, compared with in Tu‐CN mice (*Figure* 6F). Findings of these experiments suggest that SIRT6 by targeting the inhibitor WNT4 might modulate insulin secretion from pancreatic β‐cells and thereby probably curtail muscle atrophy associated with tumour growth in Tu‐Sk.T6Tg mice. In agreement with our findings, an earlier report has demonstrated that ectopic over‐expression of SIRT6 rescued defective insulin secretion from pancreatic β cells.^47^

**Figure 7:**
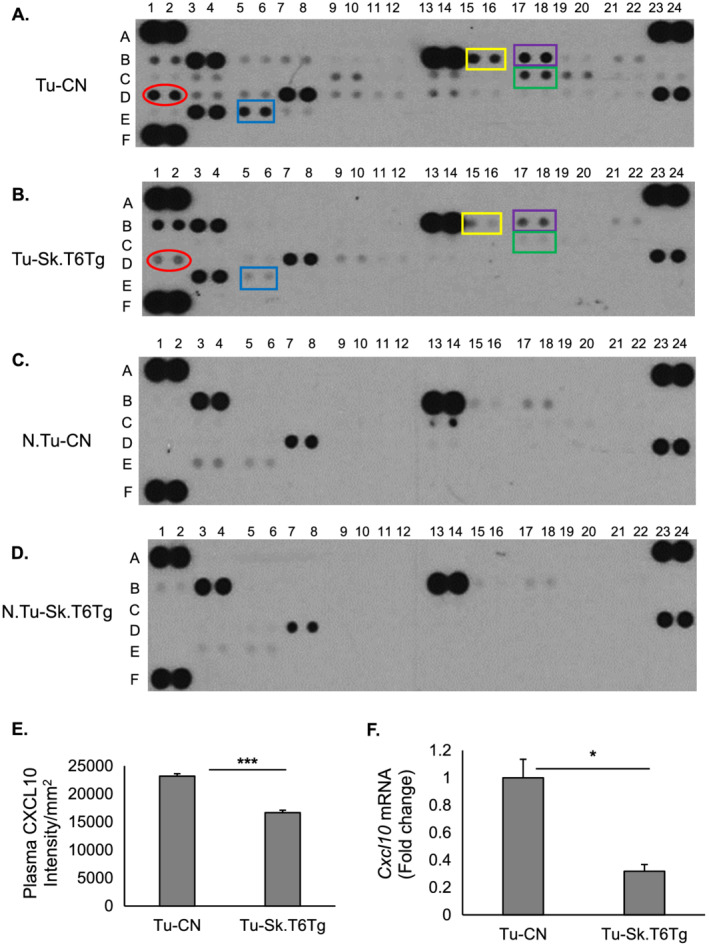
In tumour‐bearing mice, over‐expression of SIRT6 in skeletal muscles down‐regulates plasma level of CXCL10 and other cytokines associated with the pathway. Representative arrays showing results for plasma derived from (*A*) Tu‐CN, (*B*) Tu‐Sk.T6Tg, (*C*) N.Tu‐CN, and (*D*) N.Tu‐Sk.T6Tg mice. Two cytokine arrays were used for each genotype. CXCL10 is marked in red, IFN‐γ in yellow, IL‐α in purple, IL‐16 in green, and TNF‐α in blue. (*E*) Bar graph illustrating comparative intensities of CXCL10 dots obtained for Tu‐CN and Tu‐Sk.T6Tg plasma. Intensities are normalized to reference (REF) dots. In non‐tumour environment, muscle‐specific SIRT6 over‐expression by itself does not significantly alter expression levels of any cytokines in the plasma. (*F*) Bar graph showing mRNA expression levels of the chemokine *Cxcl10* in gastrocnemius muscles for the Tu‐CN and Tu‐Sk.T6Tg mice. Data represented as mean ± SEM, *n* = 5–7 mice, ***P < 0.0001, *P < 0.05.

CXCL10 is another circulating chemokine, elevated levels of which are linked to impaired pancreatic β cell function and reduced cell viability.^48^ CXCL10 is also a pro‐inflammatory biomarker identified for multiple diseases, such as cancer,^49^ diabetes,^48^ cardiovascular,^50^ and infectious diseases.^51^ CXCL10 belongs to the family of CXC chemokines and is also known as interferon‐inducible protein‐10 (IP10). It is a small 10‐kDa protein produced by many cell types, including skeletal muscle cells.^52^, ^53^ Our mouse cytokine array (Supporting Information, *Figure* S4A) analyses showed that SIRT6 over‐expression in the skeletal muscle significantly inhibited plasma levels of CXCL10 in the tumour‐bearing mice (*Figure* 7A, 7B, and 7E). However, no detectable levels of this chemokine were seen in the plasma of non‐tumour‐bearing (N.Tu‐CN, N.Tu‐Sk.T6Tg) mice (*Figure* 7C and 7D). A previous study has shown elevated serum CXCL10 levels in type 2 diabetic patients.^54^ Upregulation of CXCL10 is also linked to the development of inflammatory myopathies.^55^ Two other cytokines known to induce CXCL10 secretion from skeletal muscle cells are tumour necrosis factor (TNF)‐α and interferon (IFN)‐γ.^55^ We found that TNF‐α was significantly downregulated by SIRT6 over‐expression in tumour environment (Supporting Information, *Figure* S4B), unlike in non‐tumour background (*Figure* 7C and 7D). Other cytokines that consistently showed significant inhibition by muscle‐specific SIRT6 over‐expression in tumour settings were IL‐1α and IL‐16 (Supporting Information, *Figure* S4C and S4D). IFN‐γ showed a trend (*P* = 0.05) towards reduction in Tu‐Sk.T6Tg samples but did not cross the threshold value of significance (*P* < 0.05). In non‐tumour condition, none of the cytokines showed any statistically significant change with SIRT6 over‐expression (*Figure* 7C and 7D). As seen for plasma levels, *Cxcl10* mRNA expression was also significantly reduced in gastrocnemius muscle of Tu‐Sk.T6Tg mice, compared with that in Tu‐CN mice, indicating a direct transcriptional regulation of *Cxcl*10 by SIRT6 in the context of tumour (Figure 7F). Corroborating with our finding, an earlier study has reported increased expression of *Cxcl10* mRNA in the renal tissue of SIRT6 deficient mice.^56^ In non‐tumour‐bearing mice (N.Tu‐CN and N.Tu‐Sk.T6Tg), however, no change in *Cxcl10* transcript levels were observed (Supporting Information, *Figure* S4E) reiterating SIRT6's context‐specificity. In agreement with earlier reports,^48^, ^54^ we speculate that the SIRT6 overexpression‐associated downregulation of plasma CXCL10 level that we observe in our study could contribute to maintain the circulating insulin level in Tu‐Sk.T6Tg mice (*Figure* 4B). Based on these findings, we believe that in Tu‐Sk.T6Tg mice, over‐expressed SIRT6 might have reduced tumour growth and associated muscle wasting via downregulation of secreted CXCL10 and preservation of plasma level of insulin.

**Figure 8:**
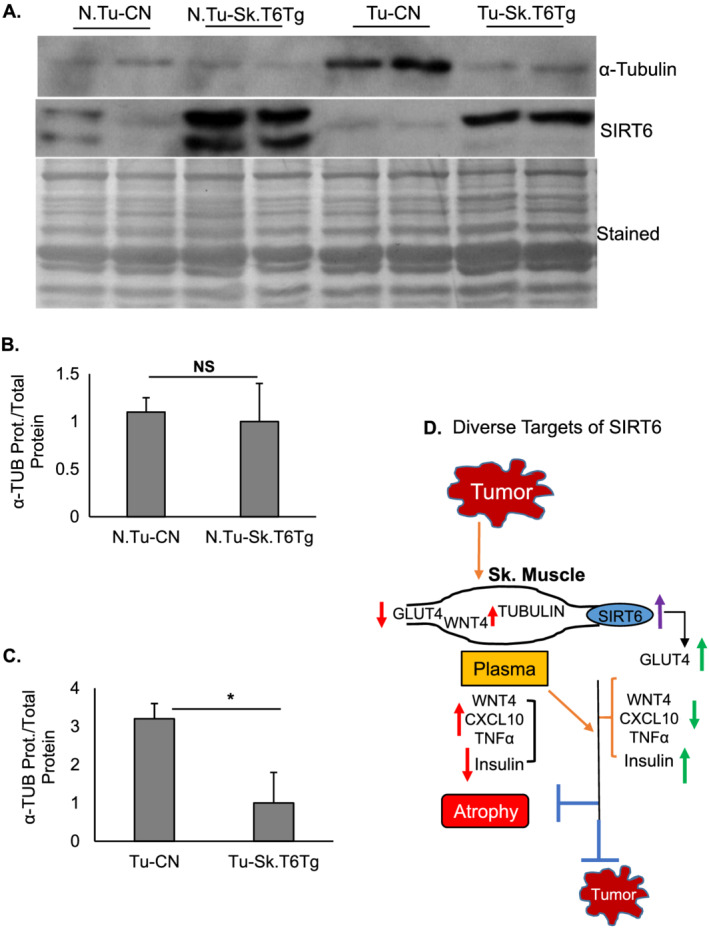
Diverse targets of SIRT6. (*A*) Tu‐Sk.T6Tg mice show downregulation of α‐TUBULIN protein expression in gastrocnemius muscle. Total protein used as a loading control. Bar graphs presenting quantitation of α‐TUBULIN protein in (*B*) non‐tumour and (*C*) tumour background for both mice categories. Data represented as mean ± SEM, *n* = 5–8 mice. NS, non‐significant, *P < 0.05. (*D*) A model illustrating different targets through which skeletal muscle‐specific SIRT6 over‐expression might be blocking tumour growth and muscle atrophy either directly or indirectly.

Apart from targeting insulin secretion, we detected yet another target of SIRT6 that might explain anti‐cachectic effect of this sirtuin. We observed that α‐TUBULIN expression was significantly downregulated in gastrocnemius muscle of Tu‐Sk.T6Tg, compared with that in Tu‐CN mice (*Figure* 8A–8C). In skeletal muscle, heterodimers of α‐ and β‐TUBULIN are needed for microtubule polymerization, and preserving a proper ratio between the two subunits is considered crucial for maintaining muscle health. Microtubule network is known to play a major role in various cellular processes.^57^ Skeletal muscle of Mdx‐mouse, a murine model of Duchenne muscular dystrophy, displays disorganized and denser microtubule cytoskeleton with dysregulated tubulins.^58^, ^59^ In hepatocellular carcinoma, increased expression of α‐TUBULIN is found to be associated with poor survival and resistance to chemotherapy.^60^ We believe that in our cancer‐cachexia model, SIRT6 over‐expression by maintaining α‐TUBULIN levels likely preserves normal microtubule organization in skeletal muscle and thereby supports muscle mass. Taken together, the data presented in this study suggest that SIRT6 has the ability to modulate multiple targets to block cancer‐induced cachexia (*Figure* 8D).

## Discussion

Skeletal muscle is a seat of major metabolic activity, and therefore, it plays a dominant role in maintenance of health of the body. Skeletal muscle is also the primary target of wasting during cachexia.^2^ Our previous study has demonstrated that by attenuating NF‐κB binding to *Mstn* promoter, SIRT6 downregulates myostatin expression and muscle loss in *in vitro* models of cachexia.^5^ In this follow‐up study, we demonstrate that muscle‐specific SIRT6 over‐expression impedes cancer‐associated muscle atrophy by targeting different pathways, including autocrine and paracrine signalling. We found that *in vivo* moderate over‐expression of SIRT6 in skeletal muscle not only inhibited myostatin expression in autocrine manner but also normalized insulin levels and downregulated secretion of non‐esterified free‐fatty acid in the plasma via paracrine signalling in tumour‐bearing mice. We also observed that the increased insulin levels were associated with reduced levels of circulating myo/chemokines, WNT4 and CXCL10, which are established inhibitors of pancreatic β‐cell function and viability.^43^, ^48^ SIRT6 over‐expression also upregulated expression of the glucose transporter, GLUT4 which is shown to improve insulin‐sensitivity of the muscle tissue and maintain the whole body glucose homeostasis.^61^ One unexpected finding of our study was that SIRT6 over‐expression helped to normalize α‐TUBULIN expression, perhaps to stabilize microtubule organization of the muscle tissue.

Due to complex biology of cachexia and its association with many chronic diseases, it has become a daunting task to identify causal factors and target them for therapies. So far, there is no single medicine or a treatment plan proven to be effective in reversing cachexia.^62^ Because multiple mechanisms are demonstrated to drive cachexia, an effective therapeutic target will be the one, which can regulate different pathways related to the cause of the disease. Insulin levels are reported to be depleted in melanoma, and systemic supplementation of insulin is shown to attenuate muscle atrophy and to reduce tumour burden.^27^ In our study, we find that SIRT6 over‐expression could restore insulin secretion to normal levels in tumour‐bearing mice. SIRT6 is a key regulator of glucose homeostasis^63^ and loss of SIRT6 in skeletal muscle is shown to impair whole body metabolism.^22^ Because many metabolic responses of skeletal muscle, such as glucose and fatty acid uptake, are mediated through insulin, its regulation by SIRT6 is an appealing strategy, not only to control tumour growth but also to limit muscle loss. In the cancer cachexia model that we used, muscle‐specific SIRT6 over‐expression was able to reduce the tumour burden in the mice. Our findings are in agreement with the earlier study, where Thackeray and colleagues have reported that systemic administration of low doses of insulin reduces tumour growth.^27^ Authors interpreted that by maintaining physiological level, insulin is re‐directed to other organs in the body such as heart and muscle, and therefore, tumour is deprived of glucose, which is the essential nutrient for tumour growth. Extrapolating from the aforementioned study, we speculate that in our cancer‐cachexia model, SIRT6‐induced increase in insulin secretion could improve glucose metabolism in the muscle, a major glucose‐consuming tissue, while depriving tumour of glucose impeding its growth. The use of insulin was also reported to decrease cancer risk in a human study in Chinese patients with type 2 diabetes.^64^ Although at this stage, we cannot comment on whether favourable effects of SIRT6 over‐expression seen on preventing muscle loss are because of restrained tumour growth in these mice, the observed dual benefits are certainly advantageous.

SIRT6 displays multiple regulatory roles and its beneficial effect is shown to be context‐dependent.^8^ While SIRT6 over‐expression is reported to cause apoptosis in many cancer cell lines that was not the case with normal untransformed cells.^65^ For the same reasons, in our study, we do not compare effects of SIRT6 over‐expression in tumour environment with those in the non‐tumour conditions. Because of SIRT6's pivotal role in maintenance of both genomic integrity and metabolic homeostasis, it is crucially important in cancer, diabetes, and other aging‐associated diseases.^66^, ^67^, ^68^ It takes on an anti‐inflammatory role by inhibiting NF‐κB signalling^13^ as well as by blocking c‐Jun‐dependent expression of pro‐inflammatory cytokines such as TNF‐α and IL‐6.^14^ Chronic systemic inflammation is the central feature of cachexia.^69^, ^70^ Our study found that SIRT6 over‐expression downregulated the pro‐inflammatory chemokine, CXCL10, which is linked, not only to functional impairment and apoptosis of β‐cells in diabetes^48^ but also to pathogenesis of cancer.^49^, ^71^ Elevated level of circulating CXCL10, as in tumour environment,^49^ can trigger β‐cell apoptosis resulting in significant reduction in insulin secretion.^48^ By reducing *Cxcl*10 mRNA expression in muscle as well as due to downregulation of circulating CXCL10 level, SIRT6 probably helps to avoid β‐cell destruction, maintaining insulin level and thereby inhibiting tumour progression.

Aberrant WNT signalling is linked with numerous pathologic conditions such as obesity, type 2 diabetes, cancer, and aging.^72^ WNT4, a member of *Wnt* family of signalling glycoproteins, is secreted specifically from muscle and adipose tissue.^43^ WNT4 is one of the 12 genes considered as a biomarker for melanoma.^73^ Kozinski *et al*. have reported that in skeletal muscle of 16 week high fat diet‐fed diabetic rats, expression of WNT4 (a canonical Wnt pathway inhibitor) was significantly increased, which correlated with drop in insulin secretion from β‐cells.^43^ Their findings also support our results, wherein we observed that in the tumour setting, SIRT6 over‐expression inhibited WNT4 expression in the skeletal muscle and downregulated circulating levels of WNT4, which might have contributed to maintain plasma insulin levels in Tu‐Sk.T6Tg mice. Furthermore, we also observed that expression of GLUT4, the principal isoform of the glucose transporter in the skeletal muscle, was upregulated in the gastrocnemius muscle of Tu‐Sk.T6Tg mice. Expression of GLUT4 is reported to be downregulated in the skeletal muscle of type 2 diabetic patients with severe insulin resistance.^34^ Muscle‐specific overexpression of GLUT4 is demonstrated to alleviate obesity and diabetes‐associated insulin resistance.^33^ Earlier evidence also reveal that increased expression of GLUT4 in skeletal muscle has beneficial effects such as lowering blood glucose and increasing insulin‐ and contractility‐associated glucose transport.^74^, ^75^ Mounting evidence suggest that SIRT6 activation provides health benefits by maintaining glucose homeostasis at multiple levels,^38^, ^42^, ^76^ and it plays a key role in promoting insulin secretion from β‐cells.^77^ In line with these earlier reports, findings of our study suggest that although SIRT6 is over‐expressed in skeletal muscle, it improves plasma insulin level in our tumour model by probably modulating multiple targets, which include CXCL10, WNT4, and GLUT4 either in paracrine and/or autocrine manner to impede the cancer‐associated cachexia.

One unpredicted finding of this study was the significant upregulation of α‐TUBULIN seen in muscle tissue of Tu‐CN mice that was downregulated by SIRT6 over‐expression. Cytoskeleton in skeletal muscle, which is made up of the dynamic grid of microtubules, is affected by physiological and pathological changes. Heterodimers of α‐ and β‐tubulin polypeptides are polymerized into microtubules, and maintenance of a proper stoichiometry between these tubulins is necessary for normal assembly. With relevance to cancer, the most widely reported microtubule changes include altered expression of tubulin isotypes.^57^ Toxic effects of altering the ratio between the two tubulins is reported in yeast.^78^, ^79^ Microtubules, tubulins, and their associated proteins regulate diverse stress responses in cells. Tubulins, in context of cancer, are targets of chemotherapeutic drugs.^57^ One proteomic study, using myoblasts from an 83‐year‐old donor, has reported significant increase in α‐TUBULIN expression when cells were treated with a cachexia‐promoting cytokine TNF‐α for 72 h.^80^ In agreement with this report, our study showed that SIRT6 over‐expression significantly downregulated plasma levels of TNF‐α, which could have led to suppression of α‐TUBULIN expression in skeletal muscle of Tu‐Sk.T6Tg mice.

In summary, findings of our study strongly suggest that skeletal muscle‐specific over‐expression of SIRT6 at a moderate level restricts tumour progression and alleviates associated cachexia by regulating diverse targets. Therefore, we believe that SIRT6 is a promising therapeutic target to treat a multifactorial syndrome like cachexia.

## Author contributions

M.P.G. and S.A.S. conceptualized this study and wrote the final draft. S.A.S. has carried out majority of the experiments, and V.B.P. helped in animal experiments.

## Conflict of interest

S.A.S., V.B.P., and M.P.G. state that there is no conflict of interest with this study.

## Supporting information


**Figure S1:** Schematic illustration describing the generation of transgenic skeletal muscle‐specific SIRT6 over‐expressing (Sk.T6Tg) mice (A, 1–4). More details about the model are given in methods' section. (B) A representative agarose gel showing genotyping for control (CN) and Sk.T6Tg mice. In PCR using tail DNA, those which yield both PCR products, one corresponding to *Rosa‐Sirt6‐Flag* (450 bp) and the other 280 bp‐long band (*Myl1‐Cre* mutant) are categorized as Sk.T6Tg mice (double positive). Wild type (WT) band of 200 bp for *Myl1‐Cre* PCR indicated *Cre* recombinase non‐expresser mice. DW: distilled water was used as a negative control, and + CN DNA positive control for the PCRs. M: 100bp DNA ladder (band of increased intensity represents 500bp).

**Figure S2**: Representative images showing morphology of gastrocnemius muscle sections in non‐tumor condition stained with hematoxylin‐eosin for (A) N.Tu‐CN and (B) N.Tu‐Sk.T6Tg mice. Scale bar: 200μm. (C) Bar graph showing gastrocnemius muscle cross‐sectional diameter and (D) fiber distribution grouped in 10μm‐apart size classes with ascending order for CN and Tg mice. Values in D are represented as percentage (%) of total number of fibers counted for the above‐ mentioned two mice categories. Data represented as mean ± SEM, *n* = 3‐5 mice per group. NS: non‐significant.

**Figure S3**: Bar graphs showing quantitation for WNT4 protein (A) N.Tu‐CN vs N.Tu‐Sk.T6Tg and for (B) Tu‐CN vs Tu‐Sk.T6Tg mice. Data represented as mean ± SEM, *n* = 5–8 mice, NS: non‐significant, *p < 0.05.

**Figure S4**: (A) The layout for mouse cytokine arrays used in this study. CXCL10 is marked in red, IFN‐γ (yellow), IL‐1α (purple), IL‐16 (green) and TNF‐α (blue). Bar graphs showing quantitation of plasma level for (B) TNF‐α (C) IL‐1α and (D) IL‐16 compared between Tu‐CN and Tu‐Sk.T6Tg mice. Two cytokine arrays were used per genotype. REF: Reference dots were used for normalization of intensities. (E) Bar graph presenting *Cxcl10* mRNA normalized to *RNA pol2* mRNA expression in gastrocnemius muscle for N.Tu‐CN vs N.Tu‐Sk.T6Tg mice. Data represented as mean ± SEM, *n* = 5–7 mice, NS: non‐significant, *p < 0.05.

Supporting Information
