# Supplementary material for: Skeletal muscle‐specific over‐expression of the nuclear sirtuin SIRT6 blocks cancer‐associated cachexia by regulating multiple targets
Source: JCSM Rapid Commun. 2020 Dec 23;4(1):40–56. doi: 10.1002/rco2.27 (PMC8237231; doi:10.1002/rco2.27)
Supplement: Supplementary file 1 — Figure S1: Schematic illustration describing the generation of transgenic skeletal muscle‐specific SIRT6 over‐expressing (Sk.T6Tg) mice (A, 1–4). More details about the model are given in methods' section. (B) A representative agarose gel showing genotyping for control (CN) and Sk.T6Tg mice. In PCR using tail DNA, those which yield both PCR products, one corresponding to Rosa‐Sirt6‐Flag (450 bp) and the other 280 bp‐long band (Myl1‐Cre mutant) are categorized as Sk.T6Tg mice (double positive). Wild type (WT) band of 200 bp for Myl1‐Cre PCR indicated Cre recombinase non‐expresser mice. DW: distilled water was used as a negative control, and + CN DNA positive control for the PCRs. M: 100bp DNA ladder (band of increased intensity represents 500bp). [file RCO2-4-40-s004.pptx]

## Slide 1
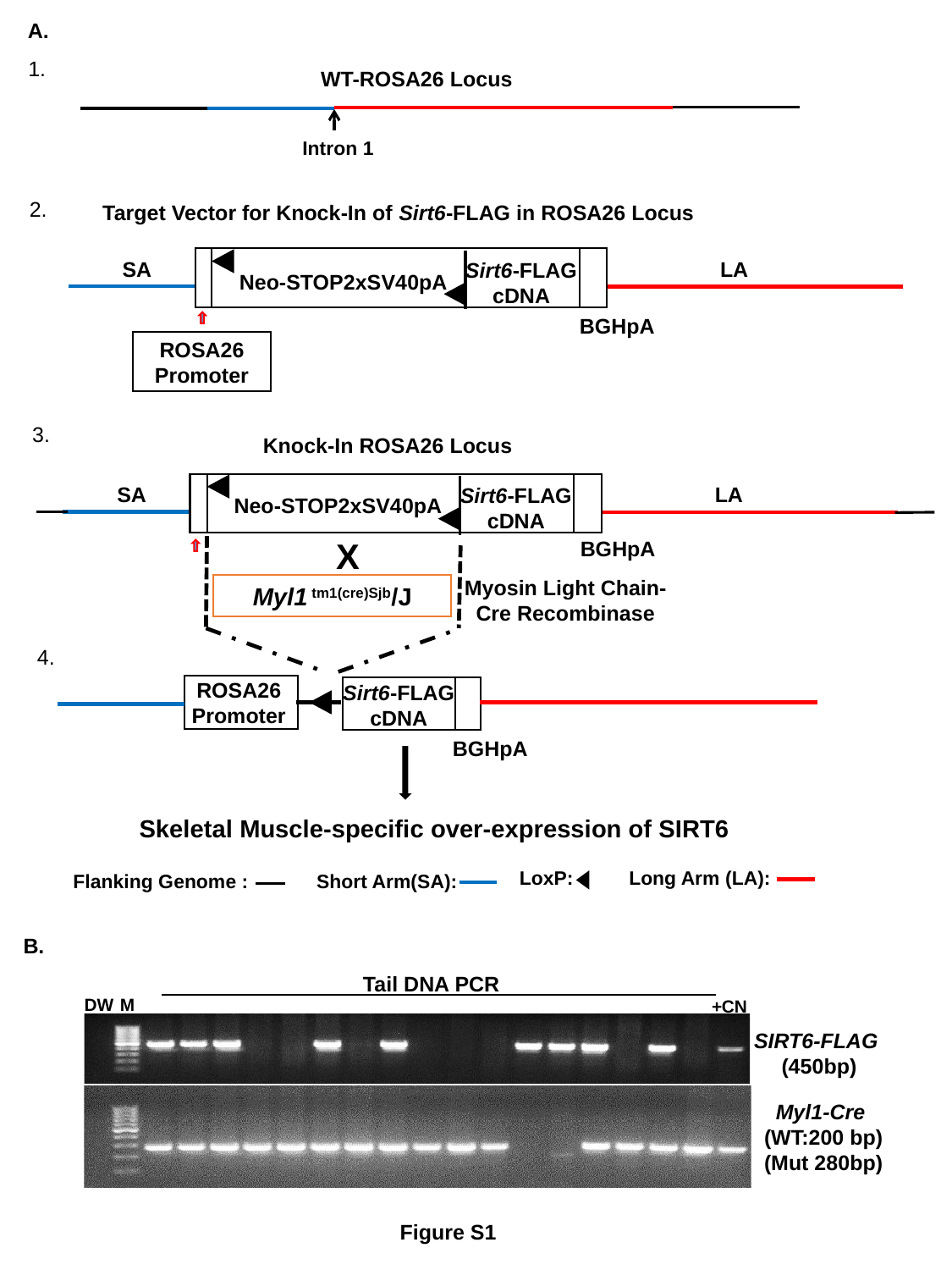

A.
1.
WT-ROSA26 Locus
Intron 1
Target Vector for Knock-In of Sirt6-FLAG in ROSA26 Locus
SA
LA
Sirt6-FLAG
cDNA
Neo-STOP2xSV40pA
BGHpA
ROSA26
Promoter
Knock-In ROSA26 Locus
SA
LA
Sirt6-FLAG
cDNA
Neo-STOP2xSV40pA
BGHpA
X
Myl1 tm1(cre)Sjb/J
ROSA26 Promoter
Sirt6-FLAG
cDNA
BGHpA
Skeletal Muscle-specific over-expression of SIRT6
LoxP:
Long Arm (LA):
Short Arm(SA):
Flanking Genome :
2.
3.
Myosin Light Chain-Cre Recombinase
4.
B.
Tail DNA PCR
DW
M
+CN
SIRT6-FLAG
(450bp)
Myl1-Cre
(WT:200 bp)
(Mut 280bp)
Figure S1
