# Supplementary material for: Skeletal muscle‐specific over‐expression of the nuclear sirtuin SIRT6 blocks cancer‐associated cachexia by regulating multiple targets
Source: JCSM Rapid Commun. 2020 Dec 23;4(1):40–56. doi: 10.1002/rco2.27 (PMC8237231; doi:10.1002/rco2.27)
Supplement: Supplementary file 2 — Figure S2: Representative images showing morphology of gastrocnemius muscle sections in non‐tumor condition stained with hematoxylin‐eosin for (A) N.Tu‐CN and (B) N.Tu‐Sk.T6Tg mice. Scale bar: 200μm. (C) Bar graph showing gastrocnemius muscle cross‐sectional diameter and (D) fiber distribution grouped in 10μm‐apart size classes with ascending order for CN and Tg mice. Values in D are represented as percentage (%) of total number of fibers counted for the above‐ mentioned two mice categories. Data represented as mean ± SEM, n = 3‐5 mice per group. NS: non‐significant. [file RCO2-4-40-s003.pptx]

## Slide 1
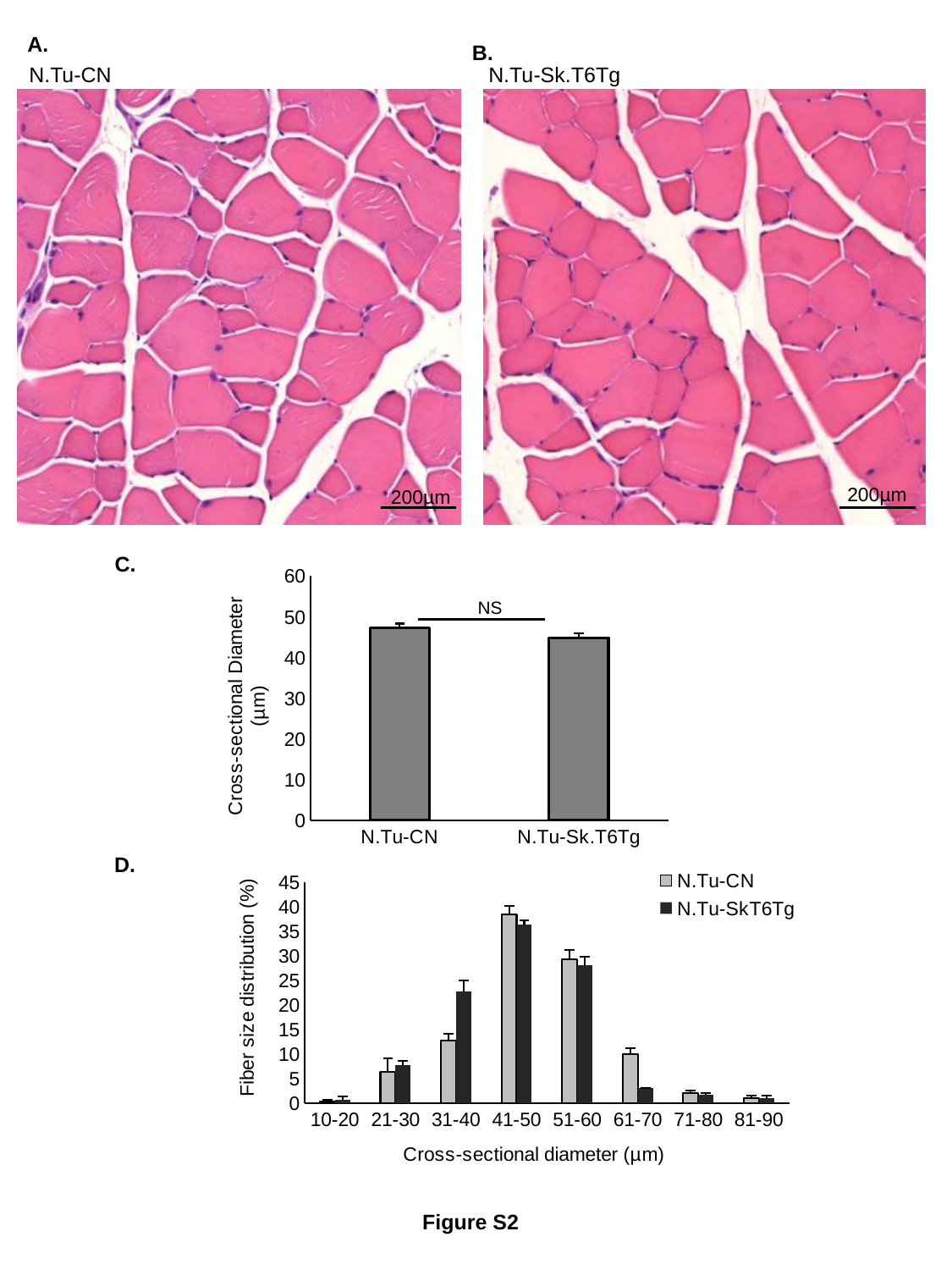

A.
B.
N.Tu-CN
N.Tu-Sk.T6Tg
200µm
200µm
C.
### Chart
| Category | Fiber size |
|---|---|
| N.Tu-CN | 47.2033644859813 |
| N.Tu-Sk.T6Tg | 44.85272727272726 |NS
D.
### Chart
| Category | N.Tu-CN | N.Tu-SkT6Tg |
|---|---|---|
| 10-20 | 0.3333333333333333 | 0.6666666666666666 |
| 21-30 | 6.333333333333333 | 7.666666666666667 |
| 31-40 | 12.666666666666666 | 22.666666666666668 |
| 41-50 | 38.333333333333336 | 36.333333333333336 |
| 51-60 | 29.333333333333332 | 28.0 |
| 61-70 | 10.0 | 3.0 |
| 71-80 | 2.0 | 1.6666666666666667 |
| 81-90 | 1.0 | 1.0 |Figure S2
