# Supplementary figures and images for: Skeletal muscle‐specific over‐expression of the nuclear sirtuin SIRT6 blocks cancer‐associated cachexia by regulating multiple targets
Source: JCSM Rapid Commun. 2020 Dec 23;4(1):40–56. doi: 10.1002/rco2.27 (PMC8237231; doi:10.1002/rco2.27)

## Slide 1
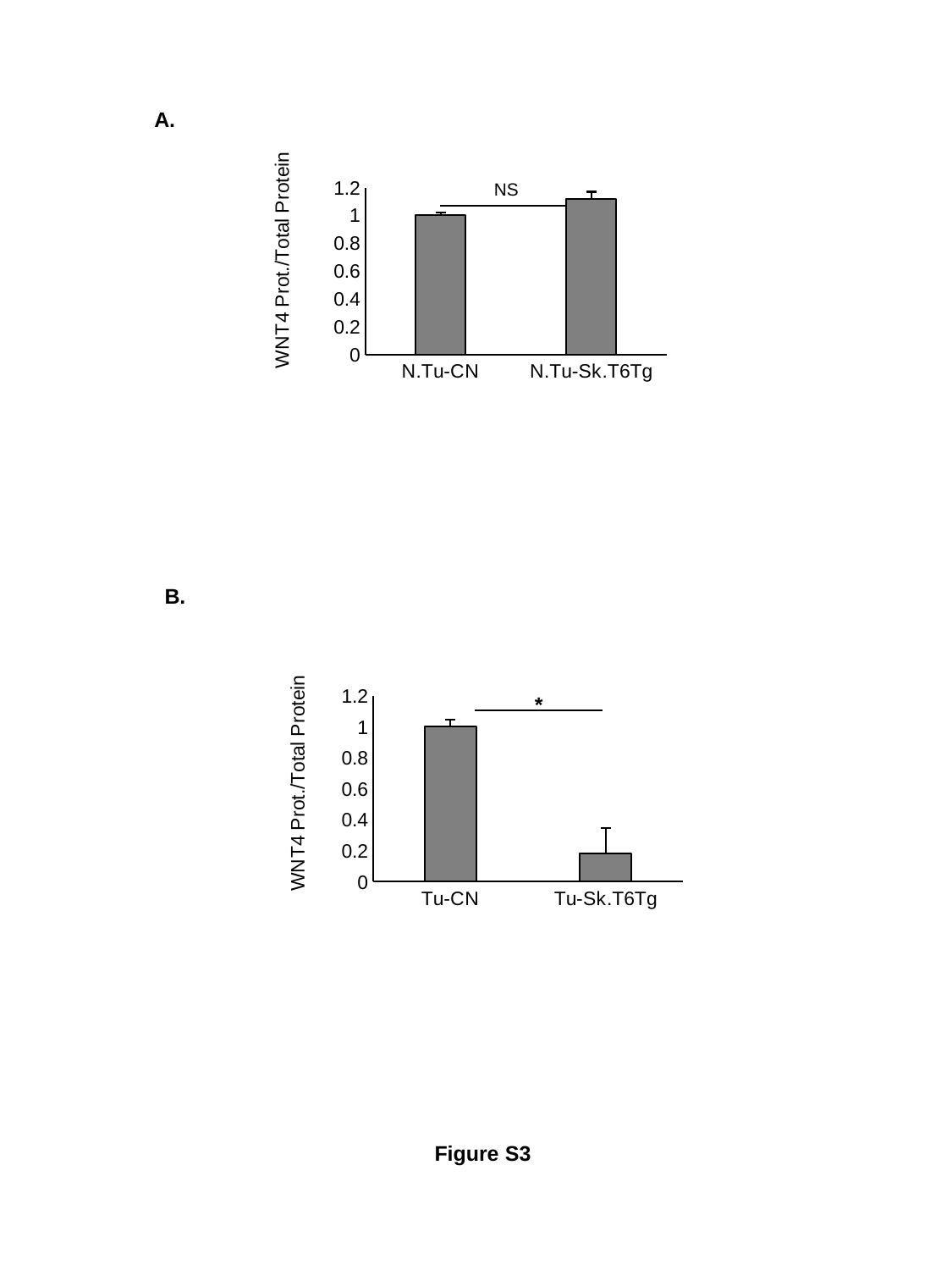

A.
### Chart
| Category | Fold |
|---|---|
| N.Tu-CN | 1.0 |
| N.Tu-Sk.T6Tg | 1.12 |NS
B.
### Chart
| Category | Fold |
|---|---|
| Tu-CN | 1.0 |
| Tu-Sk.T6Tg | 0.1826013376661957 |*
Figure S3

Supplement: Supplementary file 3 — Figure S3: Bar graphs showing quantitation for WNT4 protein (A) N.Tu‐CN vs N.Tu‐Sk.T6Tg and for (B) Tu‐CN vs Tu‐Sk.T6Tg mice. Data represented as mean ± SEM, n = 5–8 mice, NS: non‐significant, *p < 0.05. [file RCO2-4-40-s002.pptx]
