# Supplementary material for: Skeletal muscle‐specific over‐expression of the nuclear sirtuin SIRT6 blocks cancer‐associated cachexia by regulating multiple targets
Source: JCSM Rapid Commun. 2020 Dec 23;4(1):40–56. doi: 10.1002/rco2.27 (PMC8237231; doi:10.1002/rco2.27)
Supplement: Supplementary file 4 — Figure S4: (A) The layout for mouse cytokine arrays used in this study. CXCL10 is marked in red, IFN‐γ (yellow), IL‐1α (purple), IL‐16 (green) and TNF‐α (blue). Bar graphs showing quantitation of plasma level for (B) TNF‐α (C) IL‐1α and (D) IL‐16 compared between Tu‐CN and Tu‐Sk.T6Tg mice. Two cytokine arrays were used per genotype. REF: Reference dots were used for normalization of intensities. (E) Bar graph presenting Cxcl10 mRNA normalized to RNA pol2 mRNA expression in gastrocnemius muscle for N.Tu‐CN vs N.Tu‐Sk.T6Tg mice. Data represented as mean ± SEM, n = 5–7 mice, NS: non‐significant, *p < 0.05. [file RCO2-4-40-s005.pptx]

## Slide 1
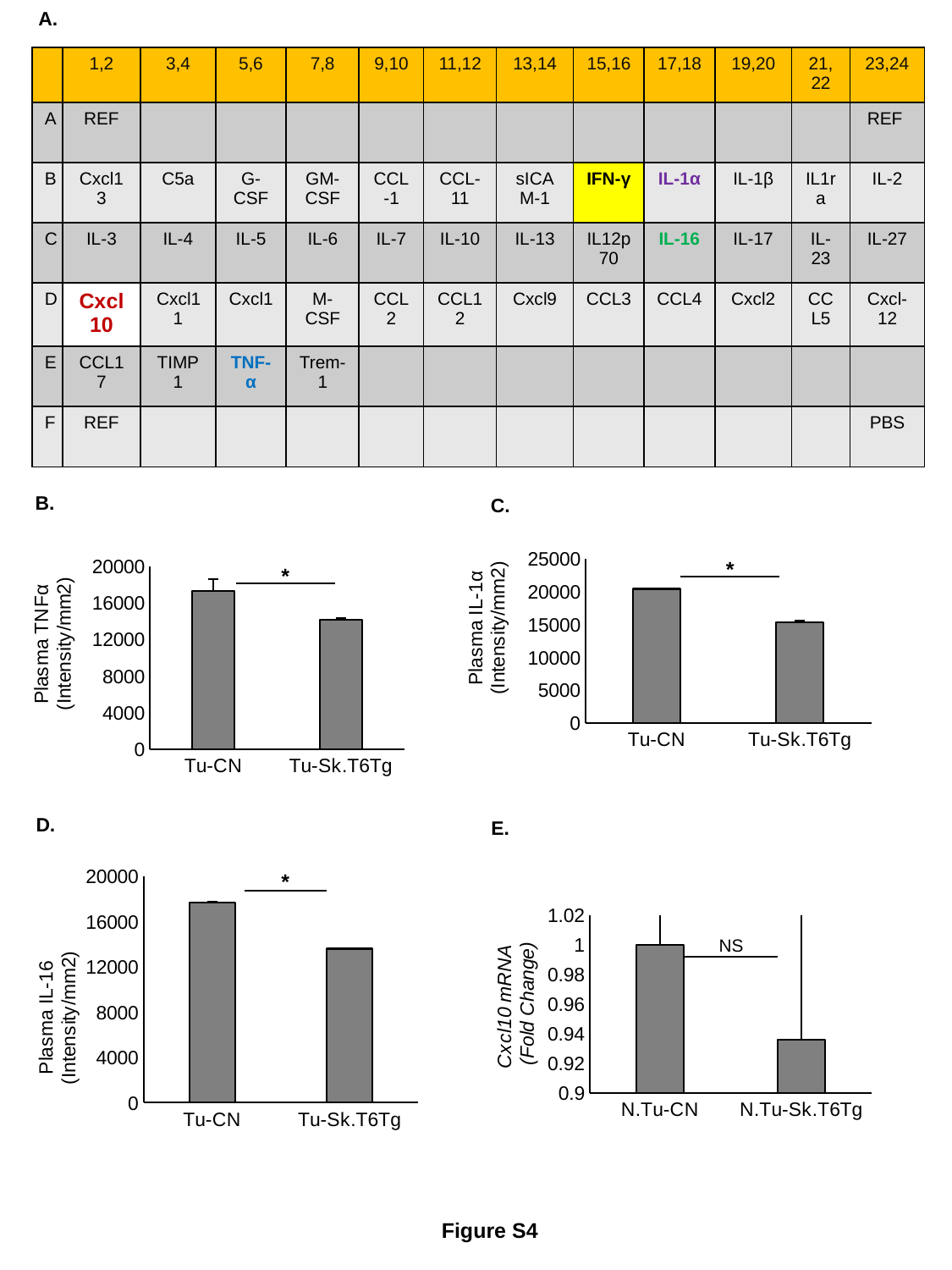

A.
| | 1,2 | 3,4 | 5,6 | 7,8 | 9,10 | 11,12 | 13,14 | 15,16 | 17,18 | 19,20 | 21,22 | 23,24 |
| --- | --- | --- | --- | --- | --- | --- | --- | --- | --- | --- | --- | --- |
| A | REF | | | | | | | | | | | REF |
| B | Cxcl13 | C5a | G-CSF | GM-CSF | CCL-1 | CCL-11 | sICAM-1 | IFN-γ | IL-1α | IL-1β | IL1ra | IL-2 |
| C | IL-3 | IL-4 | IL-5 | IL-6 | IL-7 | IL-10 | IL-13 | IL12p70 | IL-16 | IL-17 | IL-23 | IL-27 |
| D | Cxcl10 | Cxcl11 | Cxcl1 | M-CSF | CCL2 | CCL12 | Cxcl9 | CCL3 | CCL4 | Cxcl2 | CCL5 | Cxcl-12 |
| E | CCL17 | TIMP1 | TNF-α | Trem-1 | | | | | | | | |
| F | REF | | | | | | | | | | | PBS |
B.
C.
### Chart
| Category | Int |
|---|---|
| Tu-CN | 20463.959590502505 |
| Tu-Sk.T6Tg | 15325.838957010228 |
### Chart
| Category | Intensity/mm2 |
|---|---|
| Tu-CN | 17283.62758842356 |
| Tu-Sk.T6Tg | 14167.89066841171 |*
*
D.
E.
### Chart
| Category | IL16 |
|---|---|
| Tu-CN | 17661.190620127476 |
| Tu-Sk.T6Tg | 13616.061423545616 |*
### Chart
| Category | Cxcl10 |
|---|---|
| N.Tu-CN | 1.0 |
| N.Tu-Sk.T6Tg | 0.9358267023164374 |NS
Figure S4
