# Supplementary material for: Skeletal muscle‐specific over‐expression of the nuclear sirtuin SIRT6 blocks cancer‐associated cachexia by regulating multiple targets
Source: JCSM Rapid Commun. 2020 Dec 23;4(1):40–56. doi: 10.1002/rco2.27 (PMC8237231; doi:10.1002/rco2.27)
Supplement: Supplementary file 5 — Supporting Information [file RCO2-4-40-s001.docx]

**Supplemental Figure Legends:**

**Figure S1**: Schematic illustration describing the generation of transgenic skeletal muscle-specific SIRT6 over-expressing (Sk.T6Tg) mice (A, 1-4). More details about the model are given in methods’ section. (B) A representative agarose gel showing genotyping for control (CN) and Sk.T6Tg mice. In PCR using tail DNA, those which yield both PCR products, one corresponding to *Rosa-Sirt6-Flag* (450bp) and the other 280bp-long band (*Myl1-Cre* mutant) are categorized as Sk.T6Tg mice (double positive). Wild type (WT) band of 200bp for *Myl1-Cre* PCR indicated *Cre* recombinase non-expresser mice. DW: distilled water was used as a negative control, and +CN DNA positive control for the PCRs. M: 100bp DNA ladder (band of increased intensity represents 500bp).

**Figure S2**: Representative images showing morphology of gastrocnemius muscle sections in non-tumor condition stained with hematoxylin-eosin for (A) N.Tu-CN and (B) N.Tu-Sk.T6Tg mice. Scale bar: 200μm. (C) Bar graph showing gastrocnemius muscle cross-sectional diameter and (D) fiber distribution grouped in 10µm-apart size classes with ascending order for CN and Tg mice. Values in D are represented as percentage (%) of total number of fibers counted for the above- mentioned two mice categories. Data represented as mean ± SEM, n=3-5 mice per group. NS: non-significant.

**Figure S3**: Bar graphs showing quantitation for WNT4 protein (A) N.Tu-CN vs N.Tu-Sk.T6Tg and for (B) Tu-CN vs Tu-Sk.T6Tg mice. Data represented as mean ± SEM, n=5-8 mice, NS: non-significant, *p<0.05.

**Figure S4**: (A) The layout for mouse cytokine arrays used in this study. CXCL10 is marked in red, IFN-γ (yellow), IL-1α (purple), IL-16 (green) and TNF-α (blue). Bar graphs showing quantitation of plasma level for (B) TNF-α (C) IL-1α and (D) IL-16 compared between Tu-CN and Tu-Sk.T6Tg mice. Two cytokine arrays were used per genotype. REF: Reference dots were used for normalization of intensities. (E) Bar graph presenting *Cxcl10* mRNA normalized to *RNA pol2* mRNA expression in gastrocnemius muscle for N.Tu-CN vs N.Tu-Sk.T6Tg mice. Data represented as mean ± SEM, n=5-7 mice, NS: non-significant, *p<0.05.
